# Acute Psychosis Associated with Subcortical Stroke: Comparison between Basal Ganglia and Mid-Brain Lesions

**DOI:** 10.1155/2014/428425

**Published:** 2014-09-18

**Authors:** Aaron McMurtray, Ben Tseng, Natalie Diaz, Julia Chung, Bijal Mehta, Erin Saito

**Affiliations:** ^1^Neurology Division, Los Angeles Biomedical Research Institute, Torrance, CA 90502, USA; ^2^Neurology Department, Building N-25, Harbor-UCLA Medical Center, 1000 West Carson Street, Torrance, CA 90509, USA; ^3^Neurology Department, David Geffen School of Medicine, University of California at Los Angeles, Los Angeles, CA 90095, USA; ^4^Psychiatry Division, Los Angeles Biomedical Research Institute, Torrance, CA 90502, USA; ^5^Department of Psychiatry, Harbor-UCLA Medical Center, Torrance, CA 90509, USA; ^6^Department of Psychiatry and Biobehavioral Sciences, David Geffen School of Medicine, University of California at Los Angeles, Los Angeles, CA 90095, USA

## Abstract

Acute onset of psychosis in an older or elderly individual without history of previous psychiatric disorders should prompt a thorough workup for neurologic causes of psychiatric symptoms. This report compares and contrasts clinical features of new onset of psychotic symptoms between two patients, one with an acute basal ganglia hemorrhagic stroke and another with an acute mid-brain ischemic stroke. Delusions and hallucinations due to basal ganglia lesions are theorized to develop as a result of frontal lobe dysfunction causing impairment of reality checking pathways in the brain, while visual hallucinations due to mid-brain lesions are theorized to develop due to dysregulation of inhibitory control of the ponto-geniculate-occipital system. Psychotic symptoms occurring due to stroke demonstrate varied clinical characteristics that depend on the location of the stroke within the brain. Treatment with antipsychotic medications may provide symptomatic relief.

## 1. Introduction

Acute onset of hallucinations and delusions in older and elderly patients with no known history of previous psychiatric disorders should prompt a thorough investigation for secondary or neurologic causes of psychotic symptoms. Previous reports describe acute onset of psychosis resulting from acute stroke or other structural lesions affecting several different brain areas, including the prefrontal and occipital cortices, and subcortical locations such as the basal ganglia, thalamus, mid-brain, and brainstem [1, 2].

Several different mechanisms are postulated to explain acute development of psychotic symptoms due to acquired brain lesions, including direct injury to the frontal lobes or disruption of normal frontal lobe functioning through damage to connections between the prefrontal cortices and subcortical structures causing impairment of reality monitoring functions [2, 3], direct insult to primary visual cortices (Anton's syndrome) causing misinterpretation of signals from undamaged visual association cortex areas [1, 4], and loss of inhibitory control of ponto-geniculate-occipital connections leading to visual hallucinations that have been described as similar in nature to rapid eye movement (REM) sleep (peduncular hallucinosis) [2]. Consequently, we hypothesized that new onset psychosis due to acquired brain lesions may display different clinical characteristics depending on the location of the responsible lesion and the underlying brain structures and mechanisms involved.

In this report we compare two cases of new onset acute psychosis resulting from subcortical strokes located in different brain regions, the basal ganglia, and the mid-brain. The cases discussed in this report underwent brain imaging studies, general physical and neurological examinations, routine blood tests, and other studies related to workup of stroke. In both cases the psychotic symptoms developed acutely were not the result of coexistent delirium and responded well to treatment with antipsychotic medications.

## 2. Case Presentation 1: Left Basal Ganglia Hemorrhagic Stroke

A fifty-nine-year old right-handed male with no known past medical history was brought in by emergency medical services to the emergency room after acute onset at home of right sided weakness and visual and auditory hallucinations that started approximately eight hours prior to arrival. The patient was alert and oriented to self, location, and date. Examination revealed a right lower facial droop and right hemiparesis with associated pronator drift. Sensation for light touch and pin-prick was normal in the upper and lower extremities bilaterally. Deep tendon reflexes were brisk on the right side compared to the left side.

The patient reported content specific delusions that the right side of his body was “rotting,” that he had a tooth that was decaying in the right side of his mouth and that the nurses had injured the right side of his body when transporting him. Despite repeated reassurance by his treating physicians that none of these were true, he continued to display these fixed false beliefs. His visual hallucinations consisted of seeing colors and lights and hearing voices telling him that the right side of his body was “dead.” He was treated with low dose risperidone and his hallucinations steadily decreased in frequency over the course of the next two weeks.

Initial laboratory assessment showed a normal serum chemistry panel, normal complete blood cell count, normal urinalysis, and negative urine toxicology screen for illicit substances. Serum HIV testing was negative and a thyroid stimulating hormone level was within normal limits.

**Figure 1:**
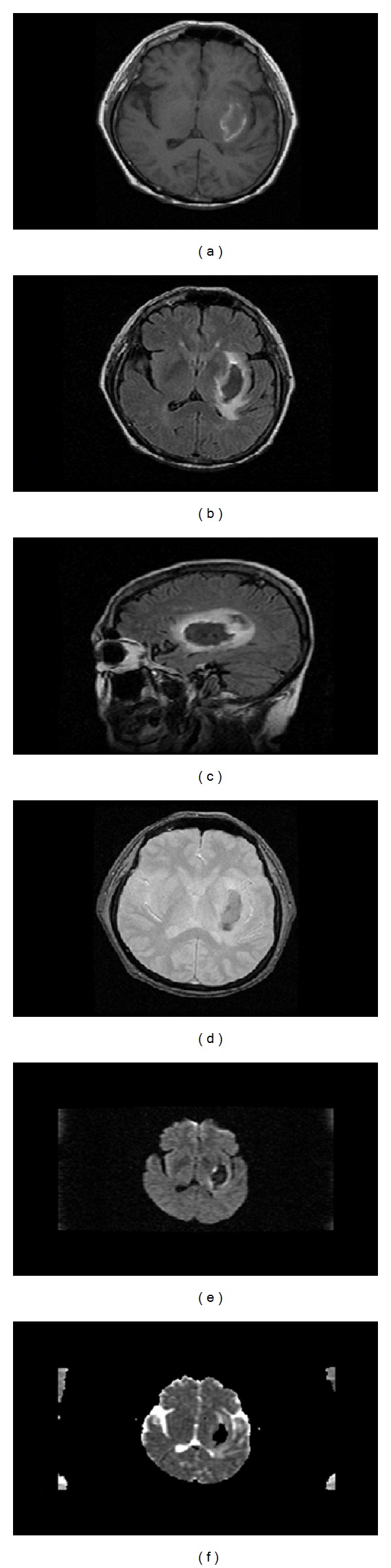
Brain magnetic resonance imaging showing a left intracranial hemorrhage. (a) Axial T1 weighted image, (b) axial T2 weighted image, (c) sagittal T1 weighted image, (d) axial gradient echo (GRE) image, (e) axial diffusion weighted image, (f) adjusted diffusion coefficient (ADC) image.

A 1.5 Tesla magnetic resonance imaging scan of the brain without contrast showed a 3.8 cm by 2.2 cm intraparenchymal hematoma located in the left basal ganglia with adjacent edema likely affecting the corona radiate and possibly extending to the optic radiations. There was no midline shift. Gray-white differentiation was preserved and the ventricles, sulci, and cisterns were normal. Additionally, no extra-axial fluid collections or significant atrophy was present, and there was no evidence of acute or subacute ischemic change. Small periventricular hyperintensities were present in the white matter on a fluid attenuated inversion recovery (FLAIR) sequence, consistent with chronic small vessel vascular disease (Figure 1).

## 3. Case Presentation 2: Peduncular Hallucinosis due to Ischemic Stroke

A fifty-two-year old right-handed female with past medical history significant for type two diabetes, hypertension, and hyperlipidemia was brought to the emergency room by a friend with new onset of dizziness and unsteadiness when walking that had developed suddenly approximately one month earlier. She also reported new onset of double vision and an occipital headache both of which had developed acutely three days prior to presentation and visual and auditory hallucinations that developed one day prior to presentation.

On presentation to the emergency room, the patient was obtunded but able to be aroused and she could answer questions and follow simple commands when aroused. The patient was noted to be grabbing at unseen objects by the nursing staff. The patient was alert and oriented to self, location, and date. Cranial nerve examination showed fixed dilated pupils bilaterally that were not reactive to light, bilateral exotropia of the eyes at rest, and complete paresis of ocular movements. The corneal and gag reflexes were present. The patient had purposeful movement in all extremities but was noted to have more spontaneous movement of the left side limbs than the right side. Sensation for light touch and pin-prick were normal in the upper and lower extremities bilaterally. Deep tendon reflexes were also normal and symmetrical throughout.

The patient's visual hallucinations were formed and consisted of seeing a deceased uncle. The patient's auditory hallucinations consisted of intermittently hearing the deceased uncle's voice saying indistinct words and sentences. The patient demonstrated preserved insight: she was aware that the hallucinations were not real and that her uncle was deceased and therefore could not be present and talk to her. The patient received a single dose of haloperidol in the emergency room due to agitation, which temporarily resolved the auditory and visual hallucinations for the remainder of that night. The patient's hallucinations were initially worse at night but then gradually decreased on scheduled haloperidol.

The initial laboratory assessment for this patient showed a normal serum chemistry panel, normal complete blood cell count, normal urinalysis, and negative urine toxicology screen for illicit substances.

**Figure 2:**
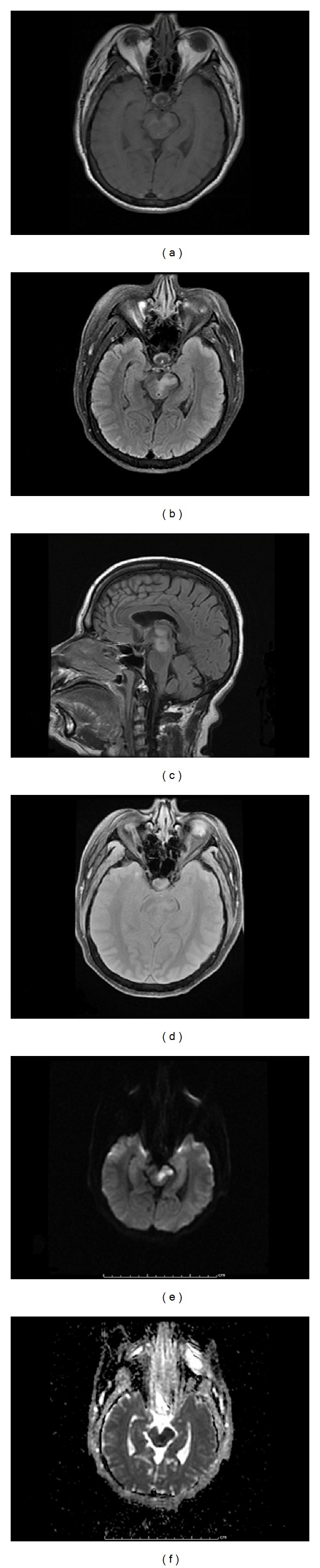
Brain magnetic resonance imaging showing acute infarcts in the thalami, left cerebral peduncle, and mid-brain. (a) Axial T1 weighted image, (b) axial T2 weighted image, (c) sagittal T1 weighted image, (d) axial gradient echo (GRE) image, (e) axial diffusion weighted image, (f) adjusted diffusion coefficient (ADC) image.

A 1.5 Tesla magnetic resonance imaging of the brain without contrast showed areas of restricted diffusion on DWI sequences located bilaterally in the thalami, left cerebral peduncle, the mid-brain, and right external capsule consistent with acute infarcts. Additional small, scattered white matter hyperintensities were present in the periventricular regions bilaterally on the FLAIR sequence, consistent with small vessel vascular disease. The ventricles, cisterns, and sulci were normal in appearance and there was no significant atrophy. There were no intra-axial or extra-axial fluid collections, no mass, and no midline shift (Figure 2).

## 4. Discussion

Psychosis is a relatively rare complication after stroke, with one large cohort study reporting a cumulative incidence of psychotic disorders of just 6.7% in the twelve years after first stroke [5]. Psychosis in patients with basal ganglia lesions is theorized to result from decreased reality testing or reality monitoring and typically manifests as a combination of content specific delusions (usually with a paranoid quality) and sometimes visual or auditory hallucinations, although these are reported less frequently [2, 3, 6]. The first case presented in this report was typical of patients who develop delusions and hallucinations due to basal ganglia lesions. The patient primarily reported fixed false beliefs related to his new physical impairment on the right side of the body, including a paranoid component as evidenced by his belief that the hospital nurses had injured him. These false beliefs were content specific and he did not exhibit delusional thinking in other areas. His visual hallucinations were unformed and his auditory hallucinations also primarily were related to the new physical impairment on the right side of his body, suggesting a content specific quality to the auditory hallucinations as well. Interestingly, the patient's lateralized nihilistic somatic delusions (that his right side was rotting and a tooth in the right side of his mouth was decaying), accompanied by auditory hallucinations telling him that the right side of his body was dead, may be consistent with Cotard syndrome (delire des negations), which has been reported in primary psychiatric disorders as well as neurologic disorders such as dementia, traumatic brain injury, and seizures [7].

Delusions and hallucinations caused by lesions in the basal ganglia are thought to occur due to disruption of normal self-corrective functions that prevent development to odd beliefs as well as impairment of the sense of familiarity which may contribute to development of paranoid ideation [3]. Normal frontal lobe functioning depends on five frontal-subcortical circuits that when damaged can alter normal behavior and contribute to development of neuropsychiatric symptoms [8]. Previous reports have demonstrated that lacunar infarction in the basal ganglia is sufficient to cause prefrontal lobe hypometabolism on positron emission tomography (PET) imaging, suggesting decreased or altered frontal lobe functioning as a direct result of the lacunar stroke [2, 9, 10]. The patients also were noted to have chronic white matter small vessel ischemic disease which could further be a disruption in the frontal subcortical pathways further facilitating the behavioral sequelae mentioned.

In the case presented in this report, the left basal ganglia lesion was quite extensive, likely causing disruption not only of the frontal subcortical circuits mentioned above but additional subcortical structures such as the external capsule, thalamus, and posterior limb of the internal capsule. This lesion was much larger for instance than the right caudate lacunar stroke lesion previously reported to cause content specific delusions [2]. Additionally, the basal ganglia lesion described in this report affected the dominant hemisphere, while the previous case report involving the caudate lacunar stroke involved the nondominant hemisphere [2]. Taking these two case reports together, it appears that unilateral basal ganglia lesions in either the dominant or nondominant hemisphere are sufficient to produce delusions [2]. This is supported by the previous case report which included functional neuroimaging data obtained from brain fluorodeoxyglucose positron emission tomography (PET) imaging in addition to the structural analysis [2]. The pervious case report used the PET imaging to determine that a unilateral lesion affecting this system can produce bilateral alterations of prefrontal functioning, which is theorized to be necessary for generation of delusions and other psychotic symptoms [2]. However, it is not currently known and has not previously been reported if unilateral prefrontal dysfunction or lesions would be sufficient to produce psychotic symptoms. Additional research utilizing advanced neuroimaging techniques such as diffusion tensor imaging to visual disruption in specific brain pathways and connections would be useful for further identifying and defining implicated pathways involved in the generation of acquired psychotic phenomena.

Peduncular hallucinosis, in contrast, was first described in the early 1920s by Jean Lhermitte [1, 4]. The pathophysiology of peduncular hallucinosis was then elicited through autopsy studies and the term “Peduncular Hallucinosis” was coined in reference to cerebral peduncles being the predominant anatomic structures thought to be involved [1, 4]. Peduncular hallucinosis is described as having a dream like quality, including vivid and colorful visual images [1, 4]. The images reported to be seen by patients with this disorder that can be scenic or bizarre are almost always formed and consist of complex objects or people [1, 4]. Lilliputian hallucinations of either animals or people have been reported as well [1, 4]. Usually there is preserved insight and the hallucinations are considered to be egosyntonic [1, 4]. Additionally there is a high percentage of hypnagogic hallucinations that predominantly occur in the evening when falling asleep and are thought to be related to a derangement of mid-brain and brainstem mechanisms that contribute to control of the sleep-wake cycle [1].

The case presented in this report displayed hallucinations typical to those reported to occur with mid-brain and thalamic injuries. Specifically, the patient had formed hallucinations with preserved insight that were worse at night, consistent with a hypnogogic component. However, there were no bizarre or Lilliputian qualities to the hallucinations, which have also been described to occur with peduncular hallucinosis.

A previous report by Benke in 2006 described auditory hallucinations in addition to visual hallucinations in a series of five cases [11]. All of the patients described by Benke reported visual and auditory hallucinations, with three of the five patients also reporting tactile hallucinations as well [11]. The auditory hallucinations described by Benke include voices, both distinct and indistinct, and sounds made by animals and in one case a train [11]. This is similar to the auditory hallucinations described by the patient in this report, who reported hearing indistinct voices from deceased relatives. However, the patient described in this report did not experience auditory hallucinations of sounds made by animals or inanimate objects such as trains, both of which were reported in some of the patients described by Benke [11]. It is not clear from the literature if involvement of certain brain structures predisposes to auditory hallucinations of voices, animals, or inanimate objects; or perhaps if personal experience and life-experience related factors play a role in the types of auditory hallucinations experienced.

The exact pathophysiology of peduncular hallucinosis remains unclear [1, 4]. The most common explanation describes peduncular hallucinosis as resulting from a release phenomenon arising from disruptions along the pathway from the ascending reticular activating system to the intralaminar thalamic nuclei [1, 4]. Others have reported cases of peduncular hallucinosis resulting from lesions primarily involving the rostral brainstem, pretectal midbrain, cerebral peduncles, substantia nigra, red nucleus, periaqueductal gray, and paramedian thalami [12–16]. Common causes beside stroke include tumors (meningiomas, primary cerebellar tumors, and metastases), subarachnoid hemorrhage, and even cases of iatrogenic injury from surgery [14, 17–20].

Taken together these cases support the idea that psychosis is a clinical syndrome that can arise from alteration of several distinct underlying brain structures and mechanisms and that clinical differences in the quality of the psychotic symptoms may reflect the particular underlying systems involved. Specifically, content specific delusions with paranoid ideation likely suggest a process disrupting normal frontal lobe reality monitoring and checking systems, while formed hallucinations with preserved insight and related to changes in the sleep cycle may suggest a release phenomenon with decreased suppression of spontaneous visual cortex functioning by brainstem and midbrain structures. Since both types of hallucinations responded well to antipsychotic medications, there is the possibility of a shared common pathway or structure necessary for psychosis to develop that responds to antidopaminergic medications.
